# Strategy insight: Mechanical properties of biomaterials’ influence on hydrogel-mesenchymal stromal cell combination for osteoarthritis therapy

**DOI:** 10.3389/fphar.2023.1152612

**Published:** 2023-04-19

**Authors:** Haoli Ying, Chengchun Shen, Ruolang Pan, Xiongfeng Li, Ye Chen

**Affiliations:** ^1^ Department of Genetics, The Children’s Hospital, Zhejiang University School of Medicine, National Clinical Research Center for Child Health, Hangzhou, China; ^2^ Department of Genetic and Metabolic Disease, The Children’s Hospital, Zhejiang University School of Medicine, National Clinical Research Center for Child Health, Hangzhou, China; ^3^ Zhejiang Provincial Key Laboratory of Genetic and Developmental Disorders, Institute of Genetics, Zhejiang University, Hangzhou, China; ^4^ Huzhou Basic and Clinical Translation of Orthopaedics Key Laboratory, Huzhou, China; ^5^ Department of Orthopedics, Huzhou Central Hospital, Zhejiang University Huzhou Hospital, Huzhou, China; ^6^ Zhejiang Provincial Key Laboratory of Cell-Based Drug and Applied Technology Development, Hangzhou, China

**Keywords:** osteoarthritis, mesenchymal stromal cells, hydrogel, cartilage regeneration, mechanical properties

## Abstract

Osteoarthritis (OA) is a kind of degenerative joint disease usually found in older adults and those who have received meniscal surgery, bringing great suffering to a number of patients worldwide. One of the major pathological features of OA is retrograde changes in the articular cartilage. Mesenchymal stromal cells (MSCs) can differentiate into chondrocytes and promote cartilage regeneration, thus having great potential for the treatment of osteoarthritis. However, improving the therapeutic effect of MSCs in the joint cavity is still an open problem. Hydrogel made of different biomaterials has been recognized as an ideal carrier for MSCs in recent years. This review focuses on the influence of the mechanical properties of hydrogels on the efficacy of MSCs in OA treatment and compares artificial materials with articular cartilage, hoping to provide a reference for further development of modified hydrogels to improve the therapeutic effect of MSCs.

## 1 Introduction

Osteoarthritis (OA) is a chronic, intractable joint disease that afflicts approximately 300 million patients worldwide (Abramoff and Caldera, 2020). Aging, traumatic joint injury, mechanical overload, and metabolic derangement are associated with an increased risk of OA. After years of basic and clinical research, OA treatment has progressed. However, the pathogenesis of OA is still unclear, and there is no cure for the degenerative disease at present. The clinical treatment methods mainly include pharmacologic therapy and surgery therapy (total joint arthroplasty), which can relieve joint pain and stiffness.

Articular cartilage degeneration is one of the most significant characteristics of OA. From the view of regenerative medicine, stem cell-induced chondrogenesis may be a promising therapeutic option. Mesenchymal stromal cells (MSCs) are a kind of pluripotent stem cells with great potential in tissue engineering. Aside from differentiating into chondrocytes, MSCs can also promote cartilage regeneration in a paracrine way. Therefore, researchers attempted to utilize MSCs and their derivative exosomes or extracellular vesicles to treat OA (Han et al., 2020; Tan et al., 2021; Jeyaraman et al., 2022; Song and Jorgensen, 2022). In recent years, clinical research strategies that combine biomaterials with MSCs to improve the efficacy of OA treatment have attracted widespread attention. A variety of hydrogels stand out due to their excellent controlled-release capability and biocompatibility. Notably, the actual effect of hydrogels is related to their mechanical properties when administered with MSCs. The mechanical properties mainly include compressive, tensile, and shear properties, which are usually examined by measuring compressive modulus, tensile modulus, and shear modulus, respectively. Optimizing the mechanical properties of hydrogels to sustain the loads in the joints is a critical issue that needs to be solved urgently.

In this review, we focus on how the mechanical properties of hydrogels influence the therapeutic efficacy of MSC-hydrogel combinations in the treatment of OA and discuss the prospects for future synthetic strategies of hydrogels.

## 2 Composition and mechanical properties of articular cartilage

Chondrocytes are the only cell type found in the articular cartilage, and they secrete large quantities of cartilaginous components at relatively early stages of cartilage development. After chondrocyte maturation, the ECM-encased cells were less dense and unevenly distributed, accounting for less than 5% of the total cartilage volume (Camarero-Espinosa et al., 2016). These mature cells have almost lost their ability to proliferate, but they can maintain compositional homeostasis in the joints through the secretome.

The body has three major categories of cartilage: fibrocartilage, elastic cartilage, and hyaline cartilage. Articular cartilage is a kind of hyaline cartilage covering the surface of joints. Cartilage tissues consist of extracellular matrix (ECM), whose main components are proteoglycans, collagen, and water (Sophia Fox et al., 2009). The synovial fluid within the articular cavity is rich in hyaluronan and proteins. Besides, it also plays a role in load support by changing its viscosity in response to external stress (Camarero-Espinosa et al., 2016).

Interestingly, the mechanical properties of cartilage depend on its anatomical position, and cartilage is regional heterogeneity. ECM tissues’ biochemical components and structure can also significantly affect intraarticular biomechanics (Fischenich et al., 2020). Due to the orthotropic material properties of articular cartilage, the measurement reported by different groups varied in methods and results. Moreover, the tested animal model should be considered because articular cartilage’s mechanical properties vary among species (Athanasiou et al., 1991). A confined compression test on bovine articular cartilage shows that the compressive aggregate modulus is between 0.08 and 2.10 MPa (Schinagl et al., 1997; Demott and Grunlan, 2022). Yet there are inconsistent values of compressive modulus measurement in joints obtained in different laboratories. For example, Bas et al. demonstrated that the compressive modulus of human knee articular cartilage is 1.63 ± 0.26 MPa (Bas et al., 2017). However, another unconfined compression test using similar experimental methods reported that the average compressive modulus of femoral cartilage is 10.60 ± 3.62 MPa at baseline (Kabir et al., 2021). It is generally agreed that the compressive modulus of the standard articular cartilage layer increases along the depth from the cartilage surface, regardless of measurements (Schinagl et al., 1997).

On the contrary, some researchers discovered that the tensile modulus and the collagen/proteoglycan ratio of the deeper articular cartilage region are lower than the superficial tissue zone (Akizuki et al., 1986). This conclusion is still debatable, as results from an indentation test suggest that the tensile modulus values of articular cartilage rise with depth, and the mean tensile modulus is 2.33 times higher than the compressive modulus (Fischenich et al., 2020). Akizuki et al. reported that most zone tensile moduli of articular cartilage ranged from 1 to 10 MPa, less than 25 MPa (Akizuki et al., 1986). The shear modulus increased with the depth of cartilage increasing, just like the compressive modulus. The surface and overall shear modulus of femoral cartilage are considerably higher than the corresponding values of tibial cartilage: the surface shear modulus of compressed femoral cartilage is 0.22 ± 0.11 MPa, and its overall shear modulus is 0.38 ± 0.06 MPa. In comparison, the value is 0.03 ± 0.003 MPa near the surface and 0.13 ± 0.01 MPa for overall cartilage obtained from the region of the lateral tibial plateau (Wong and Sah, 2010). The collagen and proteoglycan within the tissues play an important role in resisting shear stress. As people get older or OA progresses, the tensile and compressive strength of the cartilage of patients decreases. Accumulating advanced glycation end products (AGEs) and oxidative stress in joints leads to loss of articular cartilage and chondrocytes during aging, resulting in increased cartilage brittleness (Chen et al., 2002; Li et al., 2013). Accurate measurements of the aforementioned physical quantities require more feasible standard methods.

## 3 Influence of mechanical properties of hydrogels on MSC therapy for OA

Evidence suggests that material stiffness modulates MSC shape, proliferation, migration, and lineage differentiation (Selig et al., 2020). For instance, Engler et al. first reported that MSCs on stiffer substrates with elastic moduli of 25–40 kPa are committed to osteogenic lineages (Engler et al., 2006). The expression of chondrogenic marker collagen-Ⅱ on MSCs cultured on a soft matrix whose elastic modulus is about 1 kPa is significantly higher than those MSCs on a stiff substrate (Park et al., 2011). The stiffness-regulating signaling molecules involved in MSCs include RhoA/ROCK/myosin II, YAP/TAZ, TGF-β, and Wnt/β-catenin (Selig et al., 2020). Yes-associated protein (YAP) is a negative regulator of the chondrogenic differentiation of MSCs (Karystinou et al., 2015). It was demonstrated that a relatively stiff substrate induces nuclear flattening of cells seeded on it, leading to the bigger size of nuclear pores in those cells and growing YAP import into nuclei (Elosegui-Artola et al., 2017). Thus, MSCs on softer matrices tend to differentiate into chondrocytes and express less hypertrophic marker (like collagen type X) levels in 2D and 3D studies (Murphy et al., 2012; Bian et al., 2013). Hence, it is necessary to optimize the mechanical properties of hydrogels to improve therapeutic effectiveness when treated with MSCs. The mechanical properties of hydrogels are quite different because those biomaterials are based on varied macromolecular substances. Generally, hydrogels can be divided into natural materials and synthetic polymers. Natural hydrogels are broadly made from polysaccharide, protein or decellularized tissue (Catoira et al., 2019). Besides, synthetic polymers are also regarded as candidate materials for hydrogel preparation, like Polyethylene glycol (PEG), as their mechanical properties are easier to control as well as biodegradability, stability and so on. Overall, it is certain that natural articular cartilage is mechanically much stronger than most hydrogels, especially natural gels. This point of view is mainly reflected in the fact that natural articular cartilage has a higher compressive modulus or tensile modulus. Supplementary Table S1 shows some representative hydrogels and their corresponding mechanical performances.

### 3.1 Natural hydrogel

Polysaccharides, such as hyaluronic acids (HA) and chondroitin sulfate (CS), has been used clinically for their lubricating ability. A variety of hydrogels made of polysaccharides have been developed and are expected to be combined with MSCs for the treatment of OA. HA is a non-sulfated glycosaminoglycan (GAG) existing in ECM ubiquitously. HA hydrogels loaded with MSCs have been applied in treating different animal OA models (Wagenbrenner et al., 2021). 4% (v/v) HA (initial concentration is 20 mg/2 mL) hydrogels are demonstrated to promote MSC proliferation and chondrogenic gene expression of the MSC-derived chondrocytes (Wu et al., 2019). Transplantation of MSCs with HA hydrogels significantly enhanced cartilage regeneration in a porcine OA model (Wu et al., 2019). Despite HA’s relatively good mechanical properties, this type of hydrogel lacks cell adhesion motifs, so their concentration and crosslink density can limit cell spreading and proliferation. Methacrylated hyaluronic acid (HAMA) reinforces the mechanical of fibrin hydrogels slightly, and the compressive moduli of the hybrid hydrogels with 6 mg/mL fibrin varied between 3.39 ± 0.9 kPa and 6.76 ± 0.52 kPa. In contrast, the pure fibrin hydrogel has a compressive modulus of about 3.3 kPa (Snyder et al., 2014). When MSCs were cultured in fibrin/HAMA (6 mg/mL:1 mg/mL) hydrogel, it was found that the expression of *Aggrecan*, *Col2A1,* and *Sox9* was significantly upregulated, and *Col1A1* mRNA was downregulated, which show the better latent capacity of chondrogenesis and cartilage of this construct (Snyder et al., 2014).

In addition to HA, alginate, chitosan, cellulose, and many other polysaccharide-based hydrogels are widely applied in hydrogel design (Comblain et al., 2017; Bhaladhare and Das, 2022; Liu et al., 2022). Alginate incorporation into constructs enhances chondrogenesis of MSCs and tensile load bearing ability (Ma et al., 2012). The tensile modulus of pure alginate hydrogel is nearly 80 kPa, which is higher than any other alginate/fibrin blended gels (20–45 kPa) and pure fibrin hydrogel (∼30 kPa); conversely, the critical tensile strain of hybrid gels ascend on the whole as their fibrin: alginate ratios increase (Ma et al., 2012). It is also reported that the tensile moduli, the entrapped sulfated-glycosaminoglycans (GAG) and collagen content are associated with the cell density in alginate constructs containing cells increased following incubation (Williams et al., 2005). Methacrylated chitosan (MeGC) containing type II collagen (Col Ⅱ) and transforming growth factor-β1 (TGF-β1) was proved to enhance the chondrogenic ability of MSCs *in vivo,* and the incorporation of Col Ⅱ raise the compressive modulus of the construct (Choi et al., 2015). VitroGel (VG) is a kind of polysaccharide hydrogel commercially available. RGD peptide-modified VitroGel (VG-RGD) with the Dilution Solution Type 1 at the ratio of 1:1 exhibited a compressive modulus of 1.10 ± 0.13 kPa (Manferdini et al., 2022b). The value is remarkably higher than the elastic moduli of VG-3D at dilutions of 1:1 and 1:2 and VG-RGD (dilution of 1:2), which are 0.51 ± 0.10 kPa, 0.41 ± 0.09 kPa, and 0.72 ± 0.08 kPa respectively (Manferdini et al., 2022b). More interestingly, VG-RGD (1:2) favors human adipose mesenchymal stromal cell (hAMSC) differentiation into chondrocytes rather than expansion; however, no significant regulation was discovered in other VG hydrogels mentioned above (Manferdini et al., 2022b).

Protein-based scaffolds are often made of type Ⅰ collagen, which has been utilized as cartilage tissue engineering products owing to its promotion of chondrocyte proliferation, MSC chondrogenic differentiation, and adequate clinical safety (Irawan et al., 2018). Gelatin is the hydrolysate of natural collagen, and its major component is type Ⅰ collagen (Levett et al., 2014b). Gelatin and its derivatives are widely applied in regenerative medicine due to their low antigenicity and biodegradability. Gelatin methacryloyl (GelMA), a kind of chemical-modified gelatin hydrogel, is synthesized by introducing methacryloyl substitution groups onto the amine and hydroxyl groups in the gelatin chains (Van Den Bulcke et al., 2000). The photo-crosslinking induced by photoinitiator and UV irradiation leads to the formation of the GelMA network. Research groups have tested the mechanical properties of GelMA and found that its compressive modulus is positively correlated to the degree of methacrylation (Nichol et al., 2010; Chen et al., 2012). More specifically, the values of the compressive modulus of 5% (w/v) GelMA with the degree of methacryloyl substitution of 49.8%, 63.8%, and 73.2% are 2.0 ± 0.18 kPa, 3.2 ± 0.18 kPa, and 4.5 ± 0.33 kPa, respectively (Chen et al., 2012). Besides, the compressive modulus of GelMA increases with its mass/volume fraction. It was determined that the compressive moduli of 5, 10, and 15% (w/v) GelMA with a degree of methacrylation of 81.4% are about 3.3, 16.0, and 30.0 kPa. In contrast, the corresponding compressive moduli are 2.0, 10.0, and 22.0 kPa for 5, 10, and 15% (w/v) GelMA with a lower degree of methacryloyl substitution (53.8%) (Nichol et al., 2010; Yue et al., 2015). Chen et al. also observed that MSCs encapsulated and cultured in GelMA with a higher degree of methacrylation have a lower proliferative rate; however, these cells could spread and form cellular networks (Chen et al., 2012). An increase in cell density within GelMA can also lead to a decrease in the tensile modulus of the entire hydrogel, specifically, the tensile moduli of 5% (w/v) GelMA without or with cells at a concentration of 5 × 10^6^ cells/mL are 14.7 kPa and 13.3 kPa, respectively, after 96 h incubation (Krishnamoorthy et al., 2019). Because GelMA is too soft compared with articular cartilage, some research groups have developed new hybrid scaffolds or hydrogels based on GelMA and other polysaccharides to improve their mechanical properties for better application of these materials in tissue engineering.

Lin et al. developed an injectable 10% (w/v) GelMA which can crosslink fast via visible light (VL). Its stiffness and the VL exposure time present a positive correlation (Lin et al., 2014). The compressive modulus of this hydrogel with 4 min of VL exposure is 19.8 kPa. MSCs in this kind of GelMA were proved to have higher cell viability than agarose and former UV-crosslinked GelMA (Lin et al., 2014). Levett et al. found that HAMA could strengthen the mechanical properties of GelMA concentration-dependently when HAMA concentration is below 1% (w/v) (Levett et al., 2014a). The addition of 0.5% (w/v) HAMA into 9.5% (w/v) GelMA increases the compressive modulus of the cell-free hydrogel by 10–15 kPa approximately on day 1 and chondrocyte-laden hydrogel up to about 130 kPa after 8 weeks culture; besides, GelMA/HAMA has higher production and retainment of collagen type Ⅱ, GAG, and aggrecan as well as the expression of chondrogenesis-related genes compared with GelMA alone (Levett et al., 2014b). It is striking that all cell-laden GelMA/HAMA gels and cell-free GelMA/2% HAMA become stiffer after 28 and 56 days culture while the stiffness of cell-free GelMA with 0%–0.5% HAMA drops, and the amplification of compressive modulus of cell-laden hydrogels increases as the ascending of HAMA concentration (Levett et al., 2014a). In addition, Gulden et al. found that degradation of GelMA/HAMA hydrogels whose compressive moduli range from 1.5 ± 0.4 to 73.0 ± 11.1 kPa and cellular spreading in 3D constructs enhances as stiffness decreases, and HAMA could not mediate cell adhesion unless GelMA incorporation (Camci-Unal et al., 2013). Thus, if MSCs are expected to proliferate and differentiate into chondrocytes and generate sufficient cartilage matrix, the hydrogel structures must possess MSC attachment sites, and their stiffness and HA ratio should not be very high. Following this train of thought, Lin et al. synthesized a visible light-activated crosslinking hybrid scaffold composed of GelMA and HAMA at a 9%:1% (w/v) ratio; the *in vitro* experiments show its great potential for inhibiting MSC hypertrophy and promoting GAG production compared to composite hydrogels at other ratios; and researchers implanted the scaffold into a rabbit model and proved that it is capable of repairing the osteochondral defect (Lin et al., 2019). After being cultured with MSCs in a chondrogenic medium for 8 weeks, the mechanical properties of GelMA/HAMA could become stronger, and the compressive modulus of 9%:1% GelMA/HAMA is lower than GelMA with a less HA ratio (Lin et al., 2019).

Methacrylated chondroitin sulfate (CSMA) is another polysaccharide used for improving the mechanical properties of GelMA. By contrast, after cultivating with chondrocytes for the same time, the compressive modulus of GelMA/CSMA/HAMA (9%:0.5%:0.5%, w/v) hiked up from ∼35 kPa to nearly 150 kPa and increased far faster than 10% (w/v) GelMA or GelMA/CSMA (9.5%:0.5%, w/v) (Levett et al., 2014b).

Some supramolecular polymers have been used to enhance the mechanical properties of hydrogels. For instance, the combination of poly (N-acryloyl 2-glycine) (PACG) and GelMA possesses significantly higher Young’s modulus (up to 320 kPa) and sturdier tensile strength (up to 1.1 MPa) in comparison to GelMA alone (Gao et al., 2019). It has been demonstrated that the addition of bioactive glass (BG) and Mn^2+^ ions into PACG-GelMA hybrid hydrogels can benefit repair of damaged osteochondral tissues *in vivo* (Gao et al., 2019).

Gellan gum has also been incorporated into GelMA to augment the stiffness of the construct. According to the measurements attained in unconfined compression, 1% gellan gum supplement into the scaffold could elevate its Young’s modulus for about 30–40 kPa. Still, adding gellan gum reduces the cartilage matrix production by encapsulating chondrocytes with their concentration increasing (Mouser et al., 2016).

### 3.2 Synthetic hydrogel

PEG is a common material for hydrogel fabrication, but its low bioactivity and unsatisfying mechanical performance limit its application. Thus some scientists modified PEG with ECM components. For example, N-acryloyl-glucosamine (AGA) is attempted to be added into poly (ethylene glycol) diacrylate (PEGDA) to improve and synthesize glucosamine-modified PEG-based (PEG-g-GA) hydrogel finally (Yao et al., 2017). It was shown that hBMSCs within PEG-g-GA hydrogels whose AGA concentrations are 5 mM and 10 mM secrete more matrices. Their compressive modulus is mildly elevated to 39–40 kPa, surpassing other groups of different amounts of AGA supplement (Yao et al., 2017). Polyvinyl alcohol (PVA) hydrogel attracts interest for its non-toxicity, biocompatibility, and good mechanical strength. A class of hybrid hydrogels was prepared with PVA and Salecan, an extracellular-glucan. PVA/Salecan hydrogels are instrumental in contained cell adhesion. Their fracture strain ascends from 71% (pure PVA) to 84% (PVA: Salecan = 1:1, v/v) while their compressive strength and moduli descend with the increasing volume ratio of Salecan and PVA (Qi et al., 2015). Zhao et al. prepared an injectable PVA/4-carboxyphenylboronic acid (CPBA)/CaCl_2_ hydrogel which can achieve a tensile modulus of over 1 MPa and a compressive modulus of up to 5.6 MPa (Zhao et al., 2018). *In vivo* experiments confirmed that the cell-compatible construct was able to favor the formation of cartilage matrix (Zhao et al., 2018). Yan et al. also utilized DNA supramolecular hydrogel as an MSC carrier to treat severe OA rabbit models. The hydrogel/MSCs combined strategy improved efficacy in inhibiting osteophyte formation and producing regenerated cartilage similar to the normal, natural one (Yan et al., 2021). The shear-thinning behavior of this hydrogel plays a vital part in mechanical protection for MSCs encapsulated inside against shear forces (Yan et al., 2021). The shear moduli of 3D polyacrylamide (PA) hydrogel scaffolds are ranged from 1 to 12 kPa and 2D gels made of the same material have the performance of 1–121 kPa shear modulus (Hsieh et al., 2016). Harder hydrogels are proven to favor the osteogenesis of MSCs (Hsieh et al., 2016). Indeed, there are many other types of hydrogels that are mechanically comparable to natural cartilage, such as electrospun ordered fibrous membrane reinforced PVA/Polyacrylic acid (PAA)/Graphene oxide (GO) hydrogel, whose tensile and compressive modulus can reach 27.5 ± 3.2 MPa and 12.32 ± 1.35 Mpa, respectively (Chen et al., 2023). But the lack of experimental evidence keeps these hydrogels far from the criteria for clinical use.

In summary, synthetic hydrogels can provide more rigid support when hydrogels are regarded as a kind of substitute for cartilage. On the other hand, relatively softer hydrogels are more favorable for chondrogenesis by the combined use of hydrogels and MSCs. It is difficult to choose ideal hydrogels to maintain the balance between load-bearing capability and MSC affinity.

## 4 Discussion

Numerous studies have demonstrated that combining hydrogels and MSCs have great application potential in improving OA-induced cartilage defects and related joint pain. Nevertheless, the properties of hydrogels remain to be improved. Previous studies on hydrogel design and combined MSC-hydrogel therapy for OA often paid less attention to the relationship between their mechanical properties and recovery effects. In addition, data on the stiffness or viscosity of the hydrogels were lacking in some studies (Manferdini et al., 2022a). It is critical to choose hydrogels with suitable mechanical properties during the treatment of OA with different joints, varying degrees, and diverse causes. The physical variables reflecting newly developed hydrogels’ mechanical properties should be described quantitively. Here are three prospective thoughts on hydrogel design:

• Keep the balance among hydrogels’ mechanical properties, cellular affinity, and biocompatibility. Natural hydrogels are thought to be too soft to hold weight and should be modified or used with other biomaterials. Actually, hydrogels with high stiffness are not suitable for MSC proliferation and differentiation. To enhance the chondrogenesis of MSCs, the compressive moduli of hydrogels are supposed to be lower, but the optimum range remains to be further studied. Furthermore, the efficiency of MSC chondrogenesis and cartilage tissue regeneration was not satisfactory when the combination of existing hydrogels and MSCs were used for OA treatment. Balancing mechanical properties, degree of hydration of the hydrogel surface, and lubricating effect should also be considered (Zhao et al., 2018). Therefore, how to choose suitable materials to make combined therapy stents is still a research hotspot. It is known that there are three kinds of cartilage defects: osteochondral defects, full-thickness defects, and partial-thickness defects (Wei et al., 2021). The most common cartilage defect in OA joints is the last one (Guermazi et al., 2017; Wei et al., 2021). Different categories of cartilage defects vary widely in biomechanical changes. Thus, hydrogel design strategies should be devised for clinical application. The scaffold can be a composite of multiple kinds of hydrogels to imitate the anisotropy of articular cartilage, which leads to a complex mechanical process.
• Implantation of hydrogels containing MSCs may not be a panacea for OA treatment due to the complex pathology and heterogeneous symptoms of the disease. Articular cartilage repair is still the main indicator of the efficacy of osteoarthritis treatment. There are mainly two ways to treat cartilage degeneration with hydrogel: one is the transplantation of hydrogels whose mechanical properties are enough to replace damaged cartilage in OA joints, and the other is to develop hydrogels to induce cartilage regeneration and self-healing or differentiate implanted MSCs towards chondrocytes. Suppose a hydrogel is designed in the latter way of thought and degrades too quickly; in that case, its load-bearing capacity may not be a priority in scaffold design for cartilage regeneration. Coordinated application of two strategies and multiple materials may be more promising. The softer part of a hybrid hydrogel can assist MSC chondrogenic differentiation, maintenance of chondrocyte phenotype, and cartilage matrix reserve. It seems feasible to wrap the soft constructs with a more rigid synthetic polymer shell or 3D woven composite scaffolds, which play a role in bearing load (Moutos et al., 2007). The optimal hydrogel combinations with MSCs are expected to be an essential solution to OA.
