# Supplementary material for: Strategy insight: Mechanical properties of biomaterials’ influence on hydrogel-mesenchymal stromal cell combination for osteoarthritis therapy
Source: Front Pharmacol. 2023 Apr 19;14:1152612. doi: 10.3389/fphar.2023.1152612 (PMC10154526; doi:10.3389/fphar.2023.1152612)
Supplement: Supplementary file 1 [file Table1.DOCX]

Supplementary Material

Strategy insight: mechanical properties of biomaterials’ influence on hydrogel-mesenchymal stromal cell combination for osteoarthritis therapy

Haoli Ying, Chengchun Shen, Ruolang Pan, Xiongfeng Li^*^, Ye Chen^*^

*** Correspondence:** Xiongfeng Li: [lyfwind@126.com](mailto:lyfwind@126.com); Ye Chen: yechency@zju.edu.cn

# Supplementary Table

| **Hydrogel** | **Concentration** | **Seeded Cell Type** | **Modulus^*^** | **Reference** |
| --- | --- | --- | --- | --- |
| GelMA (methacryloyl substitution of 49.8%) | 5% (w/v) | MSC | 2.0 ± 0.18 kPa | (Chen et al., 2012) |
| GelMA (methacryloyl substitution of 63.8%) |  |  | 3.2 ± 0.18 kPa |  |
| GelMA (methacryloyl substitution of 73.2%) |  |  | 4.5 ± 0.33 kPa |  |
| GelMA (no cells encapsulated) | 5% (w/v) | 3T3 fibroblast | 14.7 kPa (tensile modulus, after 96 h incubation) | (Krishnamoorthy et al., 2019) |
| GelMA (5 × 10^6^ cells/mL encapsulated) | 5% (w/v) | 3T3 fibroblast | 13.3 kPa (tensile modulus, after 96 h incubation) |  |
| Engineered cartilage (made of GelMA and hBMSCs, cultured in chondrogenic medium for 28 days) | 15% | hBMSC | ~113 kPa (24 h after exposure of 0% strain impact);  ~99 kPa (24 h after exposure of 10% strain impact);  ~62 kPa (24 h after exposure of 20% strain impact);  ~65 kPa (24 h after exposure of 30% strain impact) | (He et al., 2022) |
| GelMA/HAMA | 9.5% (w/v) GelMA; 0.5% (w/v) HAMA | Chondrocyte | ~30 kPa (on day 1);  126 kPa (after 8 week-culture) | (Levett et al., 2014) |
| GelMA/HAMA | 9.5% (w/v) GelMA; 0.5% (w/v) HAMA | hBMSC | 175 kPa (after 8 week-chondrogenesis) | (Lin et al., 2019) |
|  | 9% (w/v) GelMA; 1% (w/v) HAMA |  | 150 kPa (after 8 week-chondrogenesis) |  |
| GelMA/Gallan gum | 10% (w/v) GelMA; 0.5% (w/v) Gallan | Chondrocyte | 47.2±4.1 kPa | (Mouser et al., 2016) |
| GelMA/HAMA/CSMA | 9% (w/v) GelMA; 0.5% (w/v) HAMA; 0.5% (w/v) CSMA | Chondrocyte | ~35 kPa (on day 1);  150 kPa (after 8 week-culture) | (Levett et al., 2014) |
| Sericin methacryloyl (SerMA) (the modification degrees of MA: 0.61 mmol/g) | 15% (w/v) | Chondrocyte | 4 kPa;  18 kPa (storage modulus);  14 kPa (loss modulus) | (Qi et al., 2018) |
| Sericin methacryloyl (SerMA) (the modification degrees of MA: 1.12 mmol/g) |  |  | 15 kPa;  21 kPa (storage modulus);  14 kPa (loss modulus) |  |
| Sericin methacryloyl (SerMA) (the modification degrees of MA: 1.95 mmol/g) |  |  | 36 kPa;  27 kPa (storage modulus);  14 kPa (loss modulus) |  |
| Methacrylated cartilage ECM-based hydrogel/bioink (cECM-MA) | 1% (w/v) | Goat BMSC | ~45 kPa (10% strain);  ~42 kPa (20% strain);  ~25 kPa (30% strain) | (Behan et al., 2022) |
|  | 2% (w/v) |  | ~80 kPa (10% strain);  ~75 kPa (20% strain);  ~47 kPa (30% strain) |  |
| Phenylboronic acid grafted hyaluronic acid/thiolated gelatin (HA-PBA-Gel) | 1.5% (w/v) HA-PBA; 0.5% (w/v) PVA; 1.5% (w/v) gelatin | Rabbit adipose mesenchymal stromal cell (AMSC) | 15.5 ± 0.5 kPa (compressive strength , 72% strain); 0.38-0.70 kPa (storage modulus, increase within 1 h) | (Shi et al., 2022) |
|  | 3% (w/v) HA-PBA; 1% (w/v) PVA; 1.5% (w/v) gelatin |  | 32.4 kPa (compressive strength , 72% strain); 1.83-3.37 kPa (storage modulus, increase within 1 h) |  |
| HA/PEG-LA-DM/PEGDM bioactive semi-interpenetrating network (methacrylate substitution for PEG-LA-DM is 93% and for PEGDM is 91%) | 15% (w/w) macropolymer solution (PEG-LA-DM:PEGDM=95:5, weight ratio); 5 mg/g HA (high molecular weight: 2 × 10^6^) | Bovine chondrocyte | 230 kPa | (Skaalure et al., 2014) |
|  | 15% (w/w) macropolymer solution (PEG-LA-DM:PEGDM=95:5, weight ratio); 5 mg/g HA (low molecular weight: 2.9 × 10^4^) |  | 180 kPa |  |
| Col Ⅰ hydrogel | 0.3% (w/v) | hMSC | 25 kPa; maximal stress before rupture: 14 kPa | (Valot et al., 2021) |
| 6M-2Si (silylated collagen-like peptides) | 6% (w/v) |  | 79 kPa; maximal stress before rupture: 109 kPa |  |
| 6M-2Si/6M-1Si | 6% (w/v); the molar ratio of 6M-2Si to 6M-1Si is 9:1 |  | 103 kPa; maximal stress before rupture: 119 kPa |  |
| MeGC | 2% (w/v) | Human fetal chondrocyte, SM-MSC and BMSC | 4.6 kPa | (Choi et al., 2015) |
| MeGC/Col II | 0.4% (w/v) |  | 7.0 kPa |  |
| Chitosan-graft-glycolic acid (GA)/phloretic acid (PA) (CH-GA/PA) (the degree of substitution of GA is 43, the degree of substitution of PA is 10) | 1% (w/v) | Chondrocyte | 1.3 kPa (storage modulus) | (Jin et al., 2009) |
|  | 2% (w/v) |  | 5.5 kPa (storage modulus) |  |
| SILY (a collagen-binding peptide, RRANAALKAGELYKSILYGSG)/chondroitin-6-sulfate (CS-SILY)/Col Ⅰ/ II | molar ratio of SILY to CS is 10:1 (actual: 10.08:1); 4 mg/mL total collagen | Rabbit BMSC | 201.1 ± 47.6 Pa (0.1 Hz, storage modulus);  245.6 ± 74.0 Pa (1 Hz, storage modulus) | (Kilmer et al., 2022) |
|  | molar ratio of SILY to CS is 15:1 (actual: 14.14:1); 4 mg/mL total collagen |  | 493.9 ± 87.3  Pa (0.1 Hz, storage modulus);  551.48 ± 132.3 Pa (1 Hz, storage modulus) |  |
|  | molar ratio of SILY to CS is 20:1 (actual: 18.40:1)’ 4 mg/mL total collagen |  | 684.0 ± 63.2 Pa (0.1 Hz, storage modulus);  763.5 ± 90.0 Pa (1 Hz, storage modulus) |  |
| VitroGel-RGD | 1:1(v/v,dilution) | hAMSC | 1.10 ± 0.13 kPa | (Manferdini et al., 2022) |
|  | 1:2 (v/v, dilution) |  | 0.72 ± 0.08 kPa |  |
| PEG-g-GA | PEGDA (10%, w/v); AGA (10 mmol/L) | hBMSC | 39 kPa | (Yao et al., 2017) |
|  | PEGDA (10%, w/v); AGA (5 mmol/L) |  | 40 kPa |  |
| PEG-infiltrated gelatin-hyaluronic acid (PEG + GH) | 9% (w/v) GelMA; 1% (w/v) HAMA;1-20% (w/v) PEGDA solution infiltrated | hBMSC | ~170 kPa | (Riewruja et al., 2022) |
| PVA | 10% (w/v) | COS-7 fibroblast | 145.2 ± 6.2 kPa | (Qi et al., 2015) |
| PVA/Salecan (Sal) | PVA (10%, w/v):Sal (2%, w/v)=1:9 (v/v) |  | 54.2 ± 3.4 kPa | (Qi et al., 2015) |
|  | PVA (10%, w/v):Sal (2%, w/v)=1:1 (v/v) |  | 22.8 ±1.3 kPa |  |
| DNA supramolecular hydrogel | 2.5×10^−3^ mol/L Y-scaffold; 3.75×10^−3^ mol/L linear linker | BMSC | 1.088 kPa (shear storage modulus); 0.149 kPa (shear loss modulus) | (Yan et al., 2021) |

**Supplementary Table 1.** Representative Hydrogels with their mechanical properties. *Most of the values within this column refer to the compressive moduli of corresponding hydrogels unless it is specified.
